# Prediction of physical activity and nutritional behaviors based on social cognitive theory in middle-aged population at risk of coronary artery disease in Bandar Abbas

**DOI:** 10.1038/s41598-024-75162-1

**Published:** 2024-10-24

**Authors:** Roghayeh Ezati Rad, Zahra Hosseini, Shokrollah Mohseni, Teamur Aghamolae, Marzieh Nikparvar, Mohammad Mohammadi

**Affiliations:** 1 Fertility and Infertility Research Center, Hormozgan University of Medical Sciences, Bandar Abbas, Iran; 2 Department of Health Promotion and Education, Social Determinants in Health Promotion Research Center, Hormozgan Health Institute, Hormozgan University of Medical Sciences, Bandar Abbas, Iran; 3 Social Determinants in Health Promotion Research Center, Hormozgan Health Institute, Hormozgan University of Medical Sciences, Bandar Abbas, Iran; 4 Department of Health Promotion and Education, Cardiovascular Research Center, Hormozgan University of Medical Sciences, Bandar Abbas, Iran; 5 Cardiovascular Research Center, Hormozgan University of Medical Sciences, Bandar Abbas, Iran; 6 Department of Community Medicine, Food Health Research Center Hormozgan University of Medical Sciences, Bandar Abbas, Iran

**Keywords:** Coronary artery disease, Nutritional behaviors, Physical activity, Socio-cognitive theory, Middle-aged, Health care, Risk factors

## Abstract

Coronary artery disease (CAD) is the most common cardiovascular disease and the main cause of mortality in developing countries. Since physical activity and nutritional behaviors are modifiable risk factors in people at risk of CAD, the present study aims to explore the effect of an intervention based on the social cognitive theory (SCT) on physical activity and nutritional behaviors in middle-aged population at risk of CAD in the city of Bandar Abbas. The present cross-sectional study was conducted on 519 middle-aged subjects who visited the healthcare centers in Bandar Abbas, southern Iran, in 2023. The sampling was simple randomization. The data were collected using the general physical activity questionnaire, nutritional behavior questionnaire and a questionnaire based on the social cognitive theory (SCT). Descriptive statistics were used to describe the demographic features of the sample. Pearson correlation coefficient was used to test the relationship between the variables of study. Multiple linear regression was used to test the effect of the SCT constructs on physical activity and nutrition behaviors. All statistical analyses and hypothesis testing were done in SPSS 21, at a significance level of 0.05. A total number of 519 subjects participated in this study, whose average age was 44.23 ± 7.14 years. The results of Pearson correlation test showed a statistically significant positive correlation between nutritional behaviors and the constructs of self-efficacy, collective efficacy, outcome expectations, observational learning, normative beliefs, barriers and opportunities, reinforcement and punishment, and behavioral intention. There was also a significant positive correlation between physical activity and self-efficacy, normative beliefs, social support and behavioral intention. Social support, self-efficacy, normative beliefs, observational learning, behavioral skills and knowledge were found to be the predictors of physical activity. Reinforcement and punishment, normative beliefs, collective efficacy, social support and barriers and opportunities were the predictors of nutritional behaviors in the middle-aged population. As the results of the study showed, it is suggested to increase physical activity in the middle-aged population at risk of CAD using appropriate strategies to strengthen social support through family and friends, improve self-efficacy, identify positive and negative normative beliefs. Plans should be made to improve observational learning, increase behavioral skills, and increase knowledge to improve nutritional behaviors, use appropriate strategies to provide timely and appropriate rewards and punishments, identify and strengthen positive normative beliefs, improve collective efficacy, and increase social support. To this aim, families and other individuals around the middle-aged population can help remove barriers and create opportunities.

Coronary artery disease (CAD) is the most common type of heart disease and is known as the leading cause of mortality worldwide, accounting for more than 350,000 deaths annually^1,2^. It is, in fact, the leading cause of mortality in developing countries^3^.

Hypertension, hyperlipidemia, smoking, diabetes, overweight or obesity, lack of physical activity and improper nutrition are the main risk factors of CAD^3,4^. Unhealthy eating habits and insufficient physical activity are important risk factors of CAD^5^.

Iranian people’s nutritional behavior gradually worsens with the change of healthy diets to fast and unhealthy foods and is often limited to the consumption of a few foods with no variety in diet^6,7^. In Hormozgan province, the type of residents’ nutrition is associated with the high consumption of sugary and fatty substances^8^. Research has shown that unhealthy eating habits increase in middle age^9^.

Insufficient physical activity has been known as an independent risk factor for CAD^10,11^. About 30% of the middle-aged are physically inactive^12,13^, and every year, 1.9 million people die due to inactivity^14,15^. The prevalence of low physical activity in Hormozgan province is reported to be 63.87%. This province ranks 27th in the country in terms of inactivity^16^.

Promoting a healthy lifestyle, including proper nutritional behavior and physical activity throughout life, is the best way to prevent CAD^17^. Diets rich in unsaturated fatty acids from olive oil and nuts, fruits, vegetables and fish, as defined by the Mediterranean diet, can significantly lower the risk of myocardial infarction, stroke and death from cardiovascular causes^18,19^.

The effectiveness of health education depends on the skill of using appropriate theories^20^. Theory-based interventions are more likely to succeed. The SCT can appropriately describe health behaviors such as physical activity and nutritional behaviors in relation to the interaction between the individual, environment and behavior^21^.

The SCT has helped researchers explore the key factors of health behaviors. It facilitates the knowledge of these factors to develop health interventions that lead to behavior change^22^. In the SCT theory, human behavior is described in terms of a threefold and dynamic model of causality, in which behavior, personal cognitive factors, and social-environmental influences interact, which is known as reciprocal determinism^23^ (Fig. 1). Studies based on the SCT showed that participants’ social support, self-efficacy, negative outcome expectations and self-regulation significantly affect their nutritional behavior^24^. A meta-analysis of 44 studies based on the SCT showed that the models accounted for 31% of the variance in physical activity, in which self-efficacy and intention were the most predictive^25^.

**Fig. 1:**
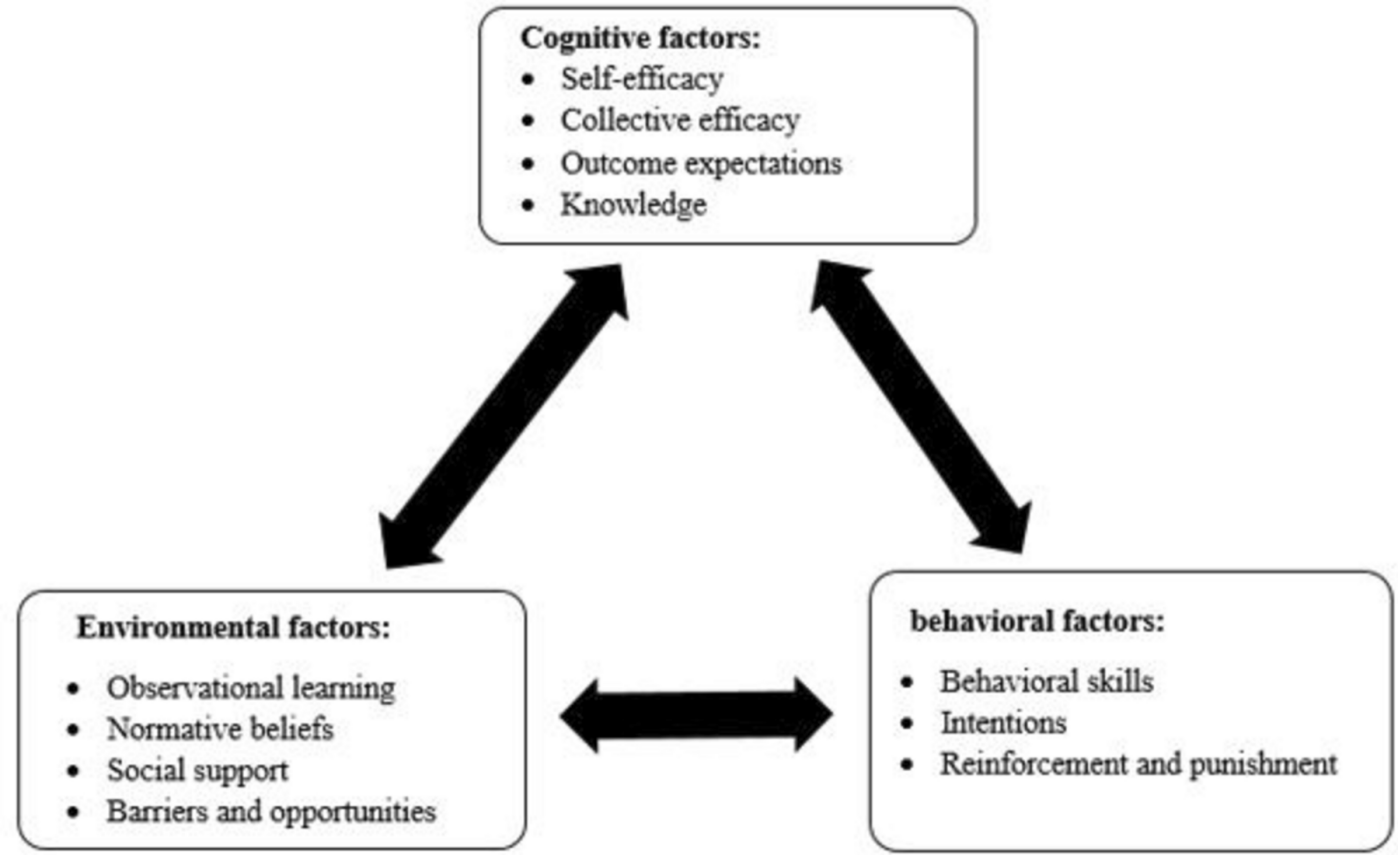
The constructs of social cognitive theory.

So far, there has been no study on how the SCT predicts physical activity and nutritional behaviors in the middle-aged at risk of CAD. Knowing the determinants of physical activity and nutritional behaviors can pave the way for planning more effective educational content to improve these two behaviors. Therefore, the present study aimed to explore the predictors of physical activity and nutritional behaviors, within the SCT, in the middle-aged at risk of CAD.

## Methods

### Design and participants

A cross-sectional study was conducted from January to May 2023 in four health centers in Bandar Abbas, in the south of Iran. The study population included all the middle-aged between 35 and 55 whose names had been listed in the National Integrated Health System (called SEEB). A total number of 519 middle-aged subjects between 35 and 55 years of age were randomly selected and phone-called. If they consented, they were met face to face to complete the questionnaire. For those who were illiterate, the answers were jotted down by the researcher.

### Sample size and sampling method

According to the multiple regression analysis, the sample size was calculated with an error of 5%, power of 80%, effect size of 0.05 and 11 constructs as the predictors in Gpower. The sample size was estimated at 346. The design effect was considered to be 1.5 and the sample size was estimated at 519. Among the comprehensive health centers in Bandar Abbas city, four were randomly selected. The 519 subjects were selected randomly from a list of middle-aged population listed in the SEEB system. Thus, 130 subjects were selected from three centers and 129 from one center. The subjects were called and invited to participate in the study. Thus, they attended the data collection meeting. A total number of 519 questionnaires were finally completed. The questionnaires were self-administered by literate participants, and were completed by the researcher for illiterate participants.

### Inclusion and exclusion criteria

The inclusion criteria were middle-age, 35–55 years of age, physical activity less than 150 min a week and at least one risk factor for CAD (i.e., hypertension, hyperlipidemia, diabetes, overweight and obesity, and tobacco consumption), having passed at least 6 months since the definitive diagnosis of the disease, no mental disease, and no heart disease. The exclusion criteria were at least three absences and failure to complete the questionnaires.

### Constructs of social cognitive theory

Self-efficacy is defined as the belief one has in oneself to successfully show the desired behaviors based on past experience or self-evaluation^26^. Collective efficacy is the belief in the ability of a group of people to perform coordinated actions to achieve an outcome^27^. Outcome expectations are also the probability of achieving a desirable outcome of showing the required behavior or perceived benefits^28^. Knowledge is the understanding of risks and benefits of various health measures and the required awareness of showing the behavior of interest^29^. Observational learning is a type of learning through which one learns new information and behaviors by observing the behavior of others and the consequences of others’ behavior^30^. Normative beliefs are cultural norms and beliefs about social acceptability and the perceived prevalence of a behavior^21^. Social support is the perceived support by important people such as family and friends in showing the behavior of interest^23,25^. Barriers and opportunities make features of the social or physical environment harder or easier to show the behavior off interest^29^. Behavioral skills are the abilities needed to successfully perform the behavior^27^. Behavioral intention is one’s decision to perform the behavior^31^. Reinforcement and punishment, giving or not giving rewards or punishment, is effective in increasing or decreasing the behavior of interest^23^.

### Instrumentation

The measurement instruments in this study were a demographic survey, Global Physical Activity Questionnaire (GPAQ), nutritional behavior questionnaire and the latest draft of a researcher-made questionnaire designed based on the SCT. The validity and reliability of these instruments were also measured and reported.

### Demographic survey

In the demographic survey, the variables of sex, age, marital status, education level, (un)employment, height and weight, accommodation, socioeconomic status, smoking, family size, and the history of CAD risk factors were included such as hypertension, diabetes, hyperlipidemia, and family history of heart attack.

### Global physical activity questionnaire (GPAQ)

There are 16 items in this questionnaire put in three groups, one measuring the physical activity an individual engages in at work, one measuring travel to and from places, and the other measuring recreational tasks. One item asks the respondent to say how many hours a day s/he is sitting. As for the reliability, the kappa coefficient of this questionnaire lies within an acceptable range (0.67–0.73) with Spearman’s coefficient of 0.67–0.81^32^. In a study in Iran, the reliability coefficient of this measurement instrument was estimated at 0.82^33^. MET was used in this instrument to measure and analyze the intensity of physical activity, found to be the ratio of the metabolic rate of work to the metabolic rate of rest. Moderate and intense physical activity, in the travel to and from work questionnaire, is estimated with an energy equivalent index (MET) of 4, 4 and 8, respectively. A physical activity range higher than 3000 MET on a weekly basis is interpreted as high (active), between 600 and 3000 MET a week as moderate (semi-active), and less than 600 MET a week as low (inactive)^34^.

### Questionnaire of physical activity and nutritional behaviors based on the SCT

This study is part of a larger project with four phases of a qualitative study, a cross-sectional instrument development and an intervention. The results of the third phase (cross-sectional study) are reported in this article. In the first phase of the project (qualitative phase), in-depth semi-structured interviews were held with 20 middle-aged subjects at risk of coronary artery disease in Bandar Abbas. It used an SCT-based qualitative content analysis approach. The items within the questionnaire were based on the results of this qualitative study. Extraction was based on the social cognitive theory ^35,36^, as described below:

There were 10 items to measure knowledge. An example is “The minimum time for effective physical activity is 10 min nonstop”. The score ranged between 0 and 10 and α = 0.860. There were 10 items to measure self-efficacy. An example is “I can eat food in any situation”. The score could range between 10 and 50 and an α of 0.859. There were 7 items to measure collective efficacy construct. An example item is “I can have healthy nutrition if supported by people around me”. The score ranged between 7 and 35 with an α of 0.899. There were 10 items to measure outcome expectations. An example item is “I will have less stress with regular physical activity”). The score could range between 10 and 50 with an α of 0.894. There were 6 items to measure observational learning. An example item is “I learn healthy nutrition from people I know”. The score range was 6–30 with an α of 0.768. There were 4 items to measure normative beliefs. An example is “People around me believe that I don’t need physical activity because I do housework”. The score in this section ranged between 4 and 20 with an α of 0.868. There were 6 items to measure social support. An example is “Those around me encourage me to do physical activity”. The score could range between 6 and 30 with an α of 0.897. There were 8 items to measure barriers and opportunities. An example is “Weather conditions have prevented me from doing physical activity”. The score could range between 8 and 40 with an α of 0.875. There were 7 items to measure reinforcement and punishment. An example is “Being responsible for my family encourages me to eat healthy food”. The score could range between 7 and 35 with an α of 0.847. There were 5 items to measure behavioral skills. An example is “If I have planning skills, I can engage in physical activity”). The score could range between 5 and 25 with an α of 0.793. There were 4 items to measure intentions. An example is “I intend to have regular physical activity”. The score could range between 4 and 20 with an α of 0.793.

### Nutrition behaviors questionnaire

The existing literature show that the Mediterranean diet plays a protective role against heart diseases. The literature particularly proves the protective effect on primary and secondary prevention of heart diseases^37–39^. The questions on Mediterranean diet asked about the use of olive oil, fruits and vegetables, meat, butter and cream, carbonated drinks, legumes, seafood, sweets, nuts, poultry and rice. There were 13 questions in the initial Mediterranean diet questionnaire, yet the item on drinking wine was omitted as it was forbidden in Islam. Also, the items on eating pork and rabbit meat were changed and the answers also altered to 5 choices that ranged from always to never. The reliability and validity of the instrument were tested and confirmed. The final version of the instrument had 10 items, an example of which is “I consume at least 4 table spoons of olive oil daily”) and 10 questions ranging between 10 and 50 with an α of 0.837.

To test the qualitative content validity, the questionnaire was given to two nutritionists, two health education experts and a cardiologist. Their feedback was used to modify the questionnaire. The test–retest method and Cronbach’s alpha were used to assess the reliability of the instrument. To this aim, 30 middle-aged subjects were asked to complete the questionnaire twice with an interval of 2 weeks. The two sets of score of questionnaire completion were used to estimate the intraclass correlation coefficient (ICC) in SPSS. The value of the ICC index was estimated at 0.886. Also, Cronbach’s alpha of the questionnaire was 0.76 before removing two questions. The reliability reached 0.837 after removing two questions.

### Ethical considerations

The present study received an approval (#IR.HUMS.REC.1401.065) from the Research Ethics Committee of Hormozgan University of Medical Sciences. The subjects were assured of the confidentiality of the data they provided. While the data were collected, those who did not wish to continue with the research were free to withdraw.

The research participants were supposed to sign a written informed consent.

### Statistical analyses

Descriptive statistics were used to check the features of the sample. Mean and standard deviation were used to describe interval variables. Frequency and relative frequency were used to describe the categorical variables.

Pearson correlation coefficient was used to test the relationship between the research variables. Multiple linear regression was used to test the effect of the SCT constructs on physical activity and nutrition behaviors. All statistical analyses and hypothesis tests were done in SPSS21, and the significance level was set at 0.05.

## Results

A total number of 1,519 middle-aged people at risk of CAD were included in the study. Most patients were female (62.6), married (81.7), held a university degree (42.2), and were housewives and employees (64%). The participants’ age ranged between 35 to 55 years with an average age of 44.23 years and a standard deviation of 7.14 years (Table 1).

**Table 1:** Research participants’ demographic information.

Demographic information	Category	f	%
Age	35–45	303	58.4
	46–55	216	41.6
Sex	Female	325	62.6
	Male	194	37.4
Marital status	Single	66	12.7
	Married	424	81.7
	Divorced	9	1.7
	Widowed	20	3.9
Employment	Employee	166	32
	Retired	41	7.9
	Housewife	166	32
	Unemployed	7	1.3
	Student	29	5.6
	Self-employed	110	21.2
Education level	Illiterate	24	4.6
	Elementary	62	11.9
	High school	98	18.9
	Diploma	116	22.4
	University	219	42.2
Economic status	Favorable	136	26.2
	Moderate	340	65.5
	Unfavorable	43	8.2
BMI	> 18.5	11	2.1
	18.5–24.9	228	43.9
	25–30	174	33.5
	< 30	106	20.4
Smoking	No	440	84.8
	Cigarettes	22	4.2
	Hookah	52	10
	Other	5	1
Hypertension	Yes	227	53.4
	No	242	46.6
Hyperlipidemia	Yes	225	43.4
	No	294	56.6
Diabetes	Yes	249	48
	No	270	52
Family history of cardiovascular diseases	Yes	156	30.1
	No	363	69.9

The participants received the highest scores in behavioral intention, collective efficacy and normative beliefs. Also, the constructs of knowledge, outcome expectations and behavioral skills received the lowest percentage of scores (Table 2).

**Table 2:** Mean, standard deviation, score range and mean percentage of SCT constructs.

Variables	Mean ± standard deviation	Maximum score	Minimum score	Range of score	Mean percentage of actual scores
Knowledge	3.52 ± 2.75	10	3	0–10	35.2
Self-efficacy	33.69 ± 6.04	33	17	10–50	67.38
Collective efficacy	25.39 ± 4.32	23	12	7–35	72.54
Outcome Expectation	19.06 ± 4.82	28	10	10–50	38.12
Observational learning	14.67 ± 3.58	20	6	6–30	48.9
Normative beliefs	13.80 ± 2.41	13	7	4–20	69
Social support	13.79 ± 3.89	20	6	6–30	45.96
Barriers and opportunities	24.60 ± 3.33	25	14	8–40	61.5
Reinforcement and punishment	16.61 ± 3.87	19	7	7–35	47.45
Behavioral skills	11.37 ± 3.07	15	5	5–25	45.48
Intentions	15.06 ± 2.45	12	8	4–20	75.3
Nutrition behavior	27.85 ± 6.19	35	15	10–50	55.7
Physical activity	208.24 ± 161.19		–	–	

**Fig. 2:**
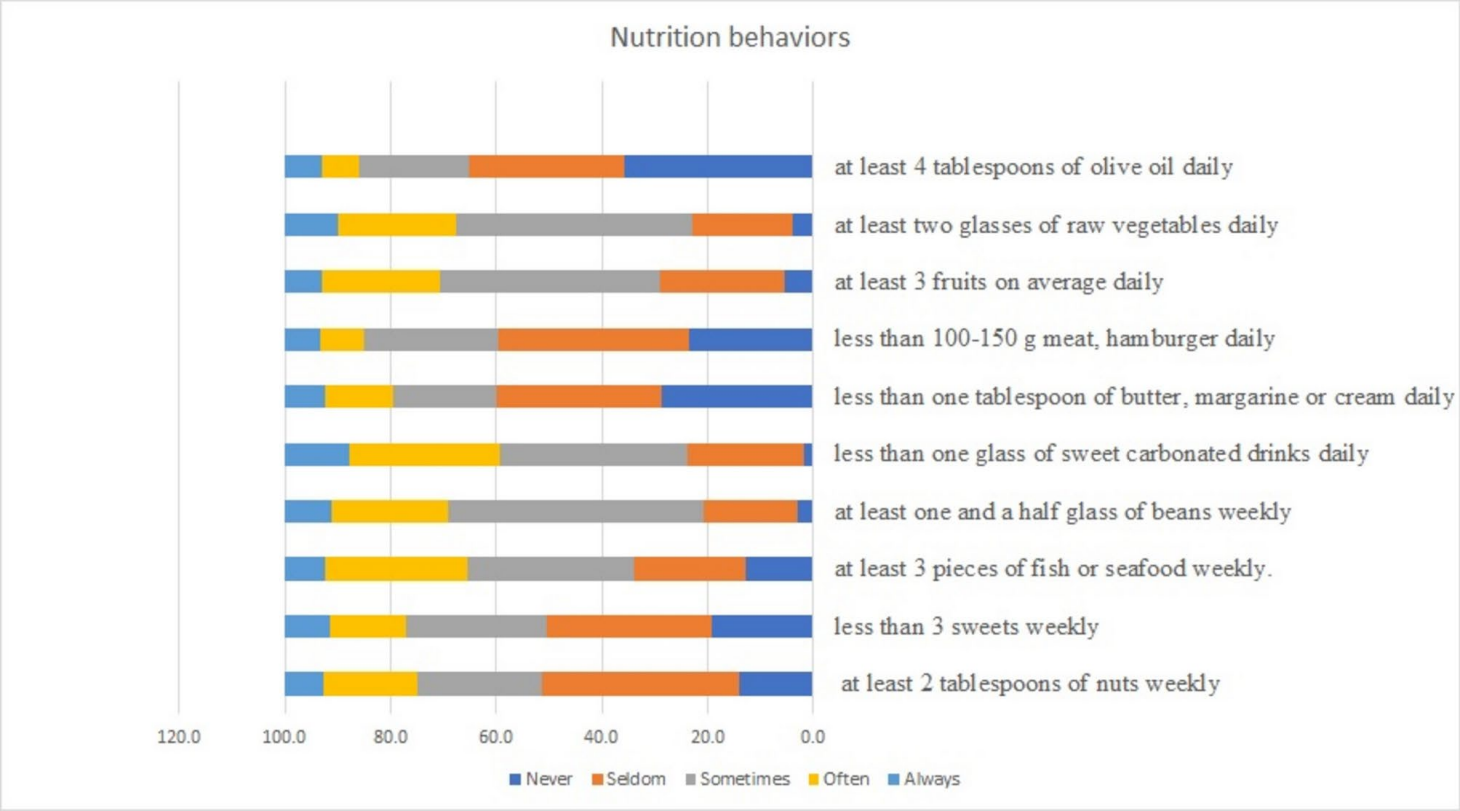
Distribution of nutrition behaviors in the participants.

The participants’ nutritional behaviors are shown in consuming liquid oil, vegetables, fruit, meat, butter, carbonated drinks, beans, seafood, sweets and nuts (Fig. 2).

**Fig. 3:**
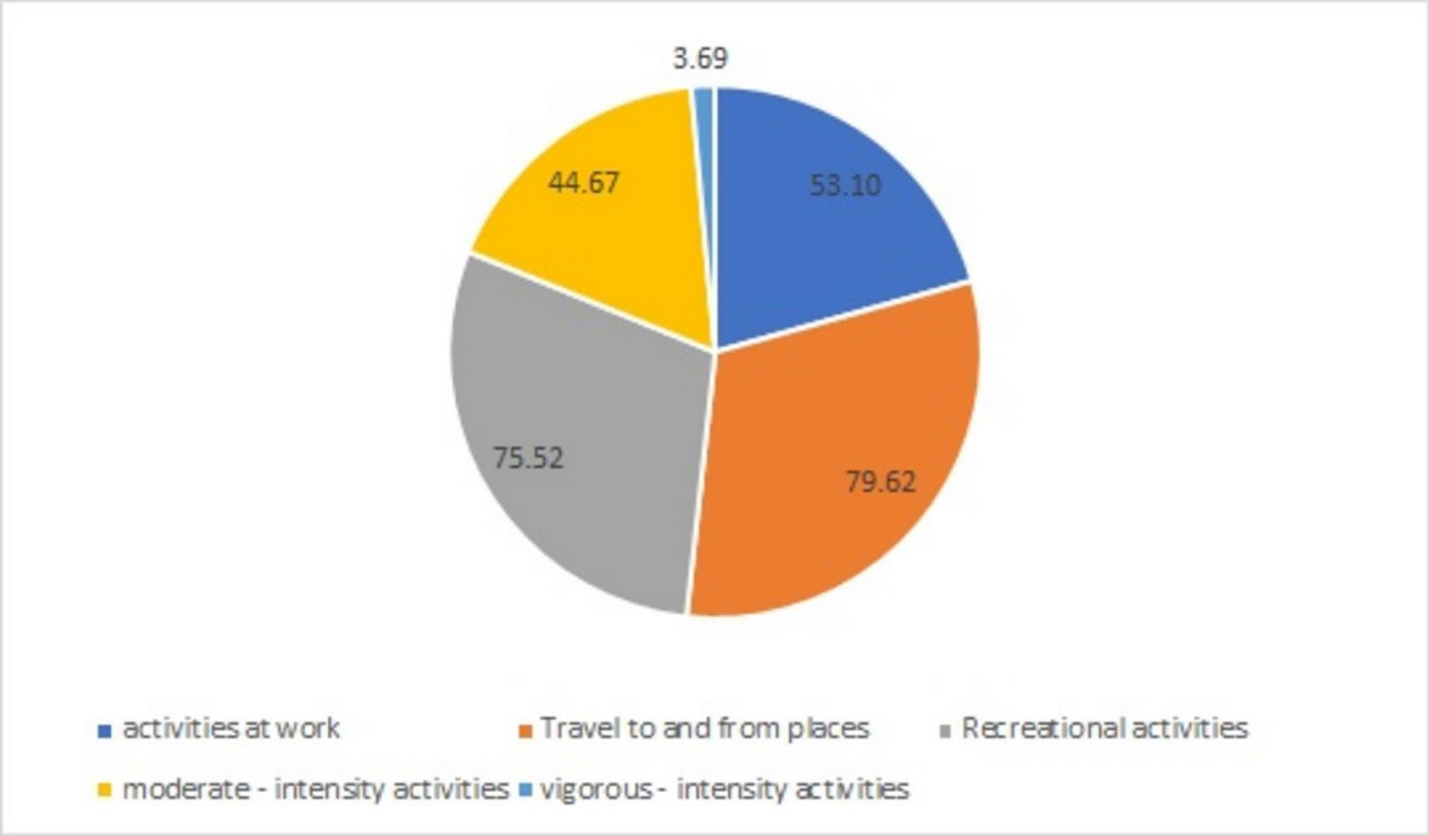
The mean score of participants’ physical activity (minutes per week).

The mean scores of participants’ physical activities per week are shown in Fig. 2, relative to their work, travel, and recreational activities, as well as moderate and intense activities. Most participants had a moderate intensity of physical activity (44.67). Also, most of their activities were in the form of movement (79.62) and recreational activities (75.52) (Fig. 3).

The results of Pearson’s correlation test showed a statistically significant positive correlation between most SCT constructs. There is also a statistically significant positive correlation between nutritional behaviors and the SCT constructs of self-efficacy, collective efficacy, outcome expectations, observational learning, normative beliefs, barriers and opportunities, reinforcement and punishment, and behavioral intention. Moreover, there is a statistically significant positive correlation between physical activity and self-efficacy constructs, normative beliefs, social support and behavioral intention (Table 3).

**Table 3:** The correlation between the constructs of the SCT.

Variables	F1	F2	F3	F4	F5	F6	F7	F8	F9	F10	F11	F12	F13
Knowledge	1												
Self-efficacy	**0.327	1											
Collective efficacy	**0.345	**0.431	1										
Outcome expectation	**0.319	**0.349	**0.353	1									
Observational learning	**0.321	**0.333	**0.607	**0.438	1								
Normative beliefs	0.047	**0.122	**0.270	**0.391	**0.286	1							
Social support	**0.389	**0.431	**0.492	**0.338	**0.503	**0/146	1						
Barriers and opportunities	**0.144	**0.378	**0.332	**0.258	**0.420	0.074	**0.401	1					
Reinforcement and punishment	**0.308	**0.304	**0.068	**0.247	**0.228	0.086	**0.321	**0.180	1				
Behavioral skills	**0.296	**0.298	**0.287	**0.426	**0.412	**0.281	**0.390	**0.210	**0.462	1			
Intentions	**0.338	**0.469	**0.384	**0.425	**0.421	**0.323	**0.438	**0.433	**0.425	**0.468	1		
Physical activity	0.004	**0.186	0.063	0.038	0.023	**0.118	**0.192	0.078	0.063	0.049	*0.1	1	
Nutrition behaviors	0.021	**0.177	**0.277	**0.165	**0.157	**0.303	0.031	**0.212	**0.140	0.065	**0.255	**0.118	1

F1 Knowledge /F2 Self-efficacy/ F3 Collective efficacy /F4 Collective efficacy /F5 Outcome Expectation / F6Observational/ learning/ F7 Social support/ F8 Barriers and opportunities/ F9 Reinforcement and punishment/ F10 Behavioral skills/ F11 Intentions/ F12 Physical activity / F13 Nutrition behavior.

The linear regression analysis showed that the constructs of social support, normative beliefs, behavioral skills, self-efficacy, observational learning and knowledge are, respectively, the main predictors of physical activity. For each unit of increase in the social support score, the physical activity score will increase for 10.027, and for each unit of increase in normative beliefs, the physical activity score will increase for 10.29. Similarly, for each unit of increase in the behavioral skill score, the physical activity score will increase for 7.750. The results showed that the given constructs explained 31% of the variance of physical activity in the middle-aged. The constructs of reinforcement and punishment, normative beliefs, behavioral intention, collective self-efficacy, social support, barriers and opportunities predict nutritional behaviors. For each unit of increase in the reinforcement and punishment score, the nutritional behaviors will increase for 0.384. For each unit of increase in the normative beliefs score, the nutritional behavior score will increase for 0.574. Similarly, for each unit of increase in the behavioral intention score, the nutritional behavior score will increase for 0.542. The results showed that the given constructs predicted 34% of the variance of nutritional behaviors in middle-aged population (Table 4).

**Table 4:** Regression analysis of the predictors of physical activity and nutritional behaviors.

Behaviors	Variable	B	Standard deviation	Standardized coefficient	T	*p*-value
Physical activity	Knowledge	6.790	2.879	0.116	2.358	0.019
	Self-efficacy	3.835	6.398	0.145	2.756	0.006
	Collective efficacy	1.37	2.2	0.037	0.623	0.534
	Outcome Expectation	2.414	1.755	0.072	1.376	0.169
	Observational learning	6.276	2.684	0.140	2.338	0.020
	Normative beliefs	10.290	3.218	0.154	3.198	0.001
	Social support	10.027	2.291	0.242	4.377	0.001
	Barriers and opportunities	1.702	2.454	0.035	0.693	0.488
	Reinforcement and punishment	1.437	2.144	0.035	0.67	0.503
	Behavioral skills	7.750	2.819	0.148	2.749	0.006
	Intentions	6.463	3.803	0.098	1.7	0.09
	Adjusted R square = 0.297					
	R square = 0.316					
Nutritional behavior	Knowledge	0.039	0.103	0.017	0.378	0.705
	Self-efficacy	0.086	0.05	0.084	1.725	0.085
	Collective efficacy	0.275	0.078	0.192	3.502	0.001
	Outcome Expectation	0.001	0.063	0.001	0.004	0.997
	Observational learning	0.095	0.096	0.055	0.998	0.319
	Normative beliefs	0.574	0.115	0.224	5.008	0.001
	Social support	0.274	0.082	0.172	3.362	0.001
	Barriers and opportunities	0.271	0.087	0.146	3.094	0.002
	Reinforcement and punishment	0.384	0.076	0.24	5.031	0.001
	Behavioral skills	0.005	0.1	0.003	0.052	0.958
	Intentions	0.542	0.136	0.215	3.996	0.001
	Adjusted R Square = 0.324					
	R-Square = 0.341					

Regression analysis of contextual and demographic variables using the backward regression method showed that the level of education (diploma) and Hookah smoking are predictors of physical activity. Moreover, Hookah smoking, masculinity, education level of below diploma, average socioeconomic status, blood pressure, and family history of heart disease are predictors of nutritional behaviors (Table 5).

**Table 5:** Regression analysis of the demographic variable predictors of physical activity and nutritional behaviors.

Behaviors	Variable	Coefficient	Std. err	t	Beta	sig	95% confidence interval	
							Lower bound	Upper bound
Physical activity	Education level (High school diploma)	40.551	17.903	2.27	0.098	0.024	5.378947	75.72387
	Smoking (Hookah)	65.445	23.336	2.8	0.122	0.005	19.599	111.290
Nutritional behavior	Smoking (Hookah)	− 2.085	0.860	− 2.42	− 0.101	0.016	− 3.776	− 0.395
	Sex male	− 1.271	0.534	− 2.38	− 0.099	0.018	− 2.322	− 0.220
	Education level illiterate	− 4.163	0.833	− 4.99	− 0.218	< 0.001	5.801	− 2.526
	Elementary	− 3.351	0.705	− 4.75	− 0.211	< 0.001	− 4.737	− 1.966
	Diploma	− 2.471	0.660	− 3.74	− 0.166	< 0.001	− 3.770	− 1.173
	Economic status Moderate	− 1.910	0.535	− 3.57	− 0.146	< 0.001	− 2.961	− 0.859
	Hypertension	− 1.220	0.513	− 2.38	− 0.098	0.018	− 2.228	− 0.212
	Family history of cardiovascular diseases	1.108	0.557	1.99	0.082	0.047	0.012	2.204

## Discussion

The present study aimed to predict physical activity and nutritional behaviors based on the SCT in middle-aged population at risk of CAD in Bandar Abbas city of Iran. The findings showed that the constructs of social support, self-efficacy, normative beliefs, observational learning, behavioral skill and knowledge were the predictors of physical activity. The constructs of reinforcement and punishment, normative beliefs, collective efficacy, social support and barriers and opportunities were the main predictors (in the SCT) of nutritional behaviors in the middle-aged.

The social support construct was the strongest predictor of doing physical activity in the middle-aged. This finding is consistent with two other studies in the literature^40,41^. Social support is the help that people receive while performing a certain behavior. Social support encourages people to adopt healthy eating habits. Social support, as the help and support by family and friends, ensures that health behaviors are adopted and managed over time^41^. A cross-sectional study was conducted on physical activity in Chinese adolescents, which showed that social support significantly affects adolescents’ physical activity in suburban areas compared to urban areas^37^. Plotnikoff et al. found that social support was highly correlated with physical activity. They emphasized that social support can significantly improve goal-setting and physical activity. Oiybo et al. have identified social support as a strong predictor of physical activity in individuals, especially women^38^.

The self-efficacy construct was another predictor of this theory for doing physical activity in the middle-aged. The higher the self-efficacy, the more likely it is to engage in physical activity. Self-efficacy plays a central and reciprocal role in predicting physical activity and is a determinant of physical activity^39^. In the study of Dewar et al. and Rahmati et al., self-efficacy was a predictor of physical activity^42,43^. In a survey by Doerksen and McAuley, it was observed that self-efficacy significantly predicts low fat consumption in eating behaviors of university employees^44^. Rolling and Hong stated in their study that high self-efficacy is related to healthy eating habits and low self-efficacy is related to unhealthy habits^45^ .

The normative beliefs construct was another predictor, in this theory, of the middle-aged performance of physical activity. The more normative beliefs are in favor of physical activity, the more likely it is to show the behavior. In another study by Baghernia et al., none of the SCT constructs were significant predictors of servings of fruits and vegetables, unhealthy foods, physical activity, and sedentary behaviors. However, normative beliefs and cultural factors played a significant predictive role^46^. Important people (also known as influential others), such as friends, may strongly affect one’s behavior^47^.

The observational learning construct was another predictor, in the SCT, of the middle-aged performance of physical activity. The findings showed that the higher the observational learning in physical activity, the more likely it is to do physical activity. Studies have shown that observational learning plays a major role in increasing physical activity^48–50^. Specialists should use peers to train and promote physical activity.

The behavioral skills construct is another predictor, in the SCT, of the middle-aged performance of physical activity. The more and better the behavioral skills, the more likely it is to do physical activity. Behavioral skills are related to physical activity and cardiovascular diseases^51^.

Knowledge was found to be a predictor of physical activity in the middle-aged, which is similar to other studies^52,53^. In their research, Kim et al. found that knowledge predicts physical activity in the disabled^53^. Knowledge supports the adoption of health-oriented behaviors and practices. Therefore, knowledge based on clear and objective information helps people adopt healthy behaviors^54^. As the present findings revealed the predictive role of knowledge in performing physical activity, providing information about physical activity as well as holding communication campaigns at the community level are essential to increase knowledge of many health-related, economic and social benefits of physical activity.

Nutritional behaviors are complex and influenced by many factors that interact at psychological, social and environmental levels^55,56^.

The findings showed that the reinforcement and punishment construct is the strongest predictor of nutritional behaviors in the middle-aged. Behavior is increased or decreased through reinforcement or punishment^57^. A relevant study showed that reinforcement and punishment predict unhealthy eating behaviors^58^. Health professionals can use psychosocial boosters to promote desirable nutritional behaviors.

The normative beliefs construct was another predictor of this theory for nutritional behaviors in the middle-aged. Normative beliefs predicted the desire to eat foods low in saturated fat. Normative beliefs play an important role in determining healthy food choices^59^. The results of Khani et al.’s study showed that normative beliefs predict nutritional behaviors related to cardiovascular diseases in women^60^.

The collective efficacy construct was another predictor, in SCT, of nutritional behaviors in the middle-aged. It implies that the greater the collective efficacy, the more likely it is to perform appropriate nutritional behaviors. In a cross-sectional study, significant relationships were found between obesity and nutritional behaviors and collective efficacy in adolescents. Moreover, there was a significant relationship between nutritional behaviors and obesity and collective efficacy with several factors, including physical and social environments, or a combination of two^61^. In Swan et al.'s study, collective efficacy, perceived price and availability of food were the main predictors of healthy eating in Dutch adults^62^.

The social support construct was also one predictor, in the SCT, of nutritional behaviors in the middle-aged. It implies that the more social support, the more likely it is to perform appropriate nutritional behaviors. Other studies have shown that low social support increases the risk of CVD^63,64^. Also, the social environment, including interaction with family, friends, and peers, affects healthy eating choices through mechanisms such as social support. It has been suggested that family members participate in educational classes together with people under intervention (patients, people at risk of CAD, etc.), because family support can be effective in maintaining the diet^65^. Oil et al.’s study highlighted the role of doctors and family support in preventing cardiovascular diseases^66^.

The barriers and opportunities construct was another predictor, in the SCT, of nutritional behaviors in the middle-aged. It means that the fewer the barriers to healthy eating and the more opportunities to seize, the more likely it is to perform the desired nutritional behaviors. In Rahmati et al.'s study, barriers predicted nutritional behaviors^43^. Teaching healthy eating behaviors, proposing models for appropriate eating habits through training sessions and providing appropriate encouragement and informative feedback in group discussions lead women to understand the benefits and barriers of right eating behaviors to prevent cardiovascular diseases. If people think they lack the required resources to perform a certain behavior, they will probably not have a strong intention to do so^60^. In a study by Rezabeigi Davarani et al., financial problems, eating habits, taste and smell were the most important barriers to the adoption of healthy eating behaviors^67^. Folta et al. found the main barriers to women’s change of behavior as the reduced risk factors of cardiovascular diseases, lack of support, different tastes and cultural and economic factors^68^. Vassallo’s study showed that perceived opportunities and barriers can improve diet^69^.

In light of the present findings, it can be concluded that the SCT is a firm theoretical framework to predict nutritional behaviors and physical activity in middle-aged population, and it is suggested to use the findings of this study to design suitable intervention programs.

### Limitations of study

The present study was conducted in an urban area and no participants from rural areas were included. Completing self-rating questionnaire and the large number of questions were the other limitations of this study. Moreover, some participants were illiterate and we faced problems in completing the questionnaires. For this purpose, we got help from their family members.

### Strengths of study

In the present study, all SCT constructs that helped explore the predictors were included. The target population was the middle-aged with different risk factors and two important and effective behaviors to prevent cardiovascular diseases (physical activity and nutritional behaviors), and these are considered the strengths of study.

## Conclusion

The present study showed that the SCT can be a good predictor of physical activity and nutritional behaviors in the middle-aged population at risk of CAD. The constructs of social support, self-efficacy, normative beliefs, observational learning, behavioral skills and knowledge were the predictors of physical activity. The constructs of reinforcement and punishment, normative beliefs, collective efficacy, social support and barriers and opportunities were among the predictors, in this theory, of nutritional behaviors in the middle-aged. For this reason, it is recommended to implement comprehensive training programs based on these results to promote physical activity and improve nutritional behavior.

## Data Availability

The datasets used and/or analyzed during the current study are available from the corresponding author on reasonable request.
